# Supplementary material for: A hybrid dense convolutional network and fuzzy inference system for pneumonia diagnosis with dynamic symptom tracking
Source: PLoS One. 2025 Oct 22;20(10):e0334899. doi: 10.1371/journal.pone.0334899 (PMC12543161; doi:10.1371/journal.pone.0334899)
Supplement: S1 Appendix — It contains S1 Appendix, which features the doctor’s comments during the creation of these fuzzy-based symptoms and conditions for pneumonia. (DOCX) [file pone.0334899.s001.docx]

**Supporting Information**

**S1 Appendix**

The Doctor’s comment during the development of fuzzy-based symptoms and conditions for pneumonia in this work are as follows:

*“While working with the team, I helped develop and verify the fuzzy rules used to classify pneumonia severity. We didn’t just pull symptoms from textbooks, but we focused on what matters during diagnosis and follow-up.”*

*“We used breathlessness, sputum production, hemoptysis, fatigue, appetite loss, confusion, cough severity, and chest pain as normalized values between 0 and 1. This approach was necessary because these symptoms can be subjective depending on the patients. Scoring them between 0 and 1 made it easier to model this uncertainty while keeping the system flexible.”*

*“We kept the fever value (°C), fever duration (days), and oxygen level (%) in their real clinical ranges. For instance, a fever of 38.3°C tells us something concrete, and converting that into 0.6 or 0.7 doesn’t make any sense.”*

*“A combination like ‘breathlessness’ is good, AND ‘fever is ‘average’ AND Oxygen level is ‘poor’ typically indicates severe pneumonia, and that’s one of the exact rules we included. We created dozens of these rules, combining different symptom categories with logic gates like AND and OR to mimic real diagnostic reasoning.”*

*“We needed these rules to cover all four levels: negligible, mild, moderate, and severe. For example, a patient with a mild cough but high fever and sputum shouldn’t be classified the same as someone with just a slight cold. That’s why we also added rule weightings; AND logic was given more weight than OR, reflecting how we think clinically.”*

*“Overall, I made sure every fuzzy rule was based on how doctors think, not just on how computers calculate.”*
