# Supplementary material for: A hybrid dense convolutional network and fuzzy inference system for pneumonia diagnosis with dynamic symptom tracking
Source: PLoS One. 2025 Oct 22;20(10):e0334899. doi: 10.1371/journal.pone.0334899 (PMC12543161; doi:10.1371/journal.pone.0334899)
Supplement: Attachment — Submitted filename: Response to Reviewers.docx [file pone.0334899.s002.docx]

**6/10/2025**

**Response to Reviewer #1:**

**Comment:** The abstract does not explain Concepts such as ‘fuzzy membership degrees’ and ‘dynamic adjustment mechanism’. Reader-friendly language should be used, or the clinical importance of the terms should be emphasized in more detail.

**Response:** Thank you for the statement. We have modified the abstract, reducing the complexity of the language, to make it reader-friendly. We have maintained PLOS one’s 300-word limit for abstract as well.

**Comment:** Demographic information (age, gender, geographical distribution), labelling process, and potential biases of the data set obtained from Kaggle are missing. It is appropriate to add these details for data quality and generalizability, which are essential for scientific studies.

**Response:** We appreciate your response regarding our selection of the dataset. We have included all possible details of the Chest X-ray dataset in the “3.1 Data Collection” part of the 3. Methodology section. We also included the link to the dataset in the supplementary material. However, the dataset doesn’t have sufficient description regarding the demographic information, so we added this issue as our limitation.

**Comment:** It is not explained how parameters such as ‘clip limit=2.0’ and ‘8x8 tile’ in CLAHE are determined. The effect of these choices on performance should be explained in detail.

**Response:** With much pleasure! We have justified our selection of those parameters via sensitivity analysis in “3.3.1.1 Contrast Limited Adaptive Histogram Equalization (CLAHE)” under the methodology section.

**Comment:** Why DenseNet201 was chosen over DenseNet121 and DenseNet169 is not analyzed in depth. This decision should be explained in detail, along with its advantages.

**Response:** Thank you for this response! This is now highlighted in the Result section under sub subheading “4.3 Results of Cross-validation methods for pneumonia classification”. There we have included Table 8, which reports Training, Testing and Validation accuracy/loss for each model and cross-validation method used.

**Comment:** The superiority of the Capsule Network over traditional CNNs is not clear. It is important to support the contribution of this method with comparative results.

**Response:** Thank you very much for this feedback! We have included a paired t-test comparison demonstrating Capsule Network’s superiority over standard CNN with a Full connection, which is provided in “4.4 Significance of capsule layers” under the Results section of our paper.

**Comment:** It is unclear how the fuzzy rules are developed with clinical experts; it should be explained in detail.

**Response:** Acknowledged! We have described the Fuzzy system under the subheading “3.3 Machine learning approach” in the Methodology section. We have also mentioned the details of an expert cardiologist in the acknowledgement section, who helped us select the pneumonia symptoms during the development of the Fuzzy rules.

**Comment:** Confidence intervals or p-values of results, such as 97.96% accuracy, are missing. Statistical robustness of the results should be demonstrated.

**Response:** Due to the large number of columns needed for 4 separate cross validation methods, initially, we only included the training metrics such as Validation loss, Validation accuracy, Test loss and Test Accuracy in “4.3 Results of Cross-validation methods for pneumonia classification”. However, we have included statistical results in “4.4 Significance of capsule layers, where we again performed a paired t-test between Capsule Layer pipeline and a standard Full connection CNN.

To avoid confusion, 99.01% is the test accuracy achieved in the original stratified k-fold based training, and 98.76% is the test accuracy achieved by further using the bootstrap sampling method for the test data (aforementioned above about the paired t-test).

**Comment:** In the article, performance evaluation is based only on accuracy. This is inadequate, especially for medical diagnostic systems, so recall, F1-score, precision, specificity, and ROC AUC should be added to the article.

**Response:** Much obliged! We mentioned our preference for DenseNet201, so we have included per-class evaluation metrics across Stratified k-folds for the DenseNet201 model on the test dataset in “4.3 Results of Cross-validation methods for pneumonia classification” under the Results section. It includes Precision, Recall, F1-Score and Specificity for each of the 5 folds via a stratified k-fold validation cross-validation method.

**Comment:** The advantages/disadvantages of the Stratified k-Fold, Bootstrap, and Monte Carlo methods and why they were chosen are not discussed. Performance differences should be analyzed in detail.

**Response:** Thank you very much for pointing this out! We have included the advantages/strengths of each method in the “3.2 Splitting the dataset using cross-validation methods” under the methodology section. Subsection “4.3 Results of Cross-validation methods for pneumonia classification” under the Results section highlights the key metrics related to the performance achieved with different DenseNet models, which indicates our selection choice.

**Comment:** How the system will be integrated in the hospital environment (e.g., compatibility with PACS) and clinician feedback are missing. A pilot study should be suggested.

**Response:** Yes, very interesting question! We have reported the future scope and direction of our work in the Conclusion and future work section of this paper.

**Response to Reviewer #2:**

**Comment:** The method section appears unnecessarily complex.

**Response:** We appreciate this observation. To improve clarity, we have revised the Methods section to streamline explanations, reduce redundancy, and more clearly distinguish the roles of each component (CNN, fuzzy inference system, and dynamic adjustment mechanism). At the beginning of the Methodology section, we have included a flow diagram summarizing the architecture to support the reader's understanding.

**Comment:** The novelty primarily lies in combining existing components rather than introducing a fundamentally new technique.

**Response:** The novelty primarily lies in combining existing components rather than introducing a fundamentally new technique.

**Comment:** We agree that some Machine Learning components in our work have already been used in previous research as well, but we emphasize that the contribution lies in their integration and clinical framing. Our system uniquely combines a state-of-the-art DenseNet201 classifier combined with Capsule Network. The latter preserves important spatial features extracted from the Deep learning procedure. Also, we developed a fuzzy inference system by noting down appropriate pneumonia-related symptoms/conditions with help from an expert cardiologist. Moreover, our work combines the confidence score generated from the DenseNet classification into the fuzzy system as a membership value. By combining it with the unique Dynamic membership adjustment algorithm, we have identified borderline cases (a case compared between the use of Deep learning-based confidence and no Deep learning-based confidence, where only one output is close to a severity level) for 4 different severity stages of pneumonia (negligible, mild, moderate and severe). This ensures clinicians effectively identify pneumonia severity stage.

**Comment:** The system’s dependence on CNN output for fuzzy logic adjustment could be more critically examined.

**Response:** Thank you for this important point! We have included a sub section: 4.5 Influence of Confidence score on pneumonia membership range, under the Results section, which highlights the difference between using CNN confidence score (also referred to as Deep learning confidence score), and without the use of such confidence score for the fuzzy inference engine. We also explained why the use of a confidence metric is essential for a proper diagnosis. However, we also include a discussion on potential limitations of this dependency in the Conclusion and future work, stating that this work of ours hasn’t been deployed in real real-world scenario yet.

**Comment:** The fuzzy rules appear to be system-defined rather than clinician-informed.

**Response:** We thank the reviewer for this concern. In the subsection 3.3.5 under Methodology, we have further made a subsection 3.3.5.1, where we reported the appropriate pneumonia guidelines used to develop the fuzzy inference system. Furthermore, this part was developed in coordination with an expert cardiologist who has been acknowledged in the respective section of our work.

**Comment:** The authors should explicitly state the study’s limitations and share their code and dataset to enhance transparency.

**Response:** We agree. We have clearly defined the limitations of our work in the section Conclusion and future work. Furthermore, we have included a proper link to the Chest X-ray dataset and the GitHub link to the relevant materials of our work in the Supplementary material section. The repository will be made public once the paper becomes fully acceptable for this journal.

**Comment:** Comment: The reviewer recommends the paper “TPat” for insights into explainable biomedical AI.

**Response:** Thank you for the suggestion. We have properly cited the paper, and explained its importance in the subsection 2.5 under the Previous Study section.
