# Supplementary material for: A hybrid dense convolutional network and fuzzy inference system for pneumonia diagnosis with dynamic symptom tracking
Source: PLoS One. 2025 Oct 22;20(10):e0334899. doi: 10.1371/journal.pone.0334899 (PMC12543161; doi:10.1371/journal.pone.0334899)
Supplement: Attachment — Submitted filename: Response_to_Reviewers_auresp_2.docx [file pone.0334899.s003.docx]

**9/08/2025**

# Response to PLOS One Editors

**Comment:** *Please include the GitHub link URL in your Data Availability Statement in the Additional Information questionnaire responses section of your submission.*

**Response:** Thank you for the comment. We have a genuine GitHub link containing all resources used in our research. Also, we have updated the Data Availability Statement of our main manuscript, containing our GitHub link. The document “Manuscript with Track Changes” has highlighted the modified section in Yellow.
Also, I have already included the link for the Dataset in the Appendix B of the supporting information section in the manuscript.
**FYI:**
Github: <https://github.com/sulav1995/Pneumonia-Diagnosis-with-ML-and-Fuzzy>
Dataset: https://www.kaggle.com/datasets/moazeldsokyx/chest-x-ray-images-pneumonia-dataset

# Miscellaneous

The respective Orcid ID of the researchers are as follows:
Sulav Baral: <https://orcid.org/0009-0005-2073-9499>

Rabindra Bista: <https://orcid.org/0000-0002-0638-5840>

Sanjog Sigdel: <https://orcid.org/0000-0002-0470-860X>

João C Ferreira: https://orcid.org/0000-0002-6662-0806
